# The Effect of Negative Pressure on Wound Healing and Regeneration in Closed Incisions under High Tension: Evidence from Animal Studies and Clinical Experience

**DOI:** 10.3390/jcm12010106

**Published:** 2022-12-23

**Authors:** Hui-Yi Hsiao, Wei-Chuan Hsieh, Frank Chun-Shin Chang, Jia-Wei Liu, Wen-Ling Kuo, David Chon-Fok Cheong, Jung-Ju Huang

**Affiliations:** 1 Center for Tissue Engineering, Chang Gung Memorial Hospital, Linkou Medical Center, Taoyuan 333, Taiwan; 2 Division of Reconstructive Microsurgery, Department of Plastic and Reconstructive Surgery, Chang Gung Memorial Hospital, Linkou Medical Center and College of Medicine, Chang Gung University, Taoyuan 333, Taiwan; 3 Division of Craniofacial Surgery, Department of Plastic and Reconstructive Surgery, Chang Gung Memorial Hospital and College of Medicine, Chang Gung University, Taoyuan 333, Taiwan; 4 Division of Breast Surgery, Department of Surgery, Chang Gung Memorial Hospital, Linkou Medical Center and College of Medicine, Chang Gung University, Taoyuan 333, Taiwan; 5 Breast Cancer Center, Chang Gung Memorial Hospital, Linkou Medical Center, Taoyuan 333, Taiwan; 6 School of Medicine, National Tsing Hua University, Hsinchu 300, Taiwan

**Keywords:** negative pressure, tensioned wound healing, profunda artery perforator flap, breast reconstruction

## Abstract

Closed-incision negative-pressure wound therapy (iNPWT) is known to enhance wound healing and tissue regeneration. The main aim of the present study is to investigate its effectiveness on enhancing wound healing under tension. An animal study was designed using a swine model by removing a skin flap to create a wound that could be closed primarily under tension, and iNPWT was applied. The enhancement of angiogenesis, lymphangiogenesis, collagen deposition, and tissue proliferation with reduced inflammation by iNPWT was confirmed by histology. The effect of iNPWT was further verified in patients receiving a profunda artery perforator (PAP) free flap for breast reconstruction. iNPWT was applied on the transversely designed donor site in continuous mode for 7 days, in which the wound was always closed under tension. A significant improvement in off-bed time was noted with the application of iNPWT (4.6 ± 1.1st and 5.5 ± 0.8th postoperative days in the iNPWT and control groups, respectively, *p* = 0.028). The control group (without iNPWT treatment) presented more cases of poor wound healing in the acute (23.1% vs. 0%) and wound breakdown in the late (23.1% vs. 8.3%) stages. The treatment of closed incisions under tension with iNPWT clinically enhances wound healing and tissue regeneration and with histological evidence.

## 1. Introduction

Negative-pressure wound therapy (NPWT), a mechanical therapy, has been reported to promote the healing of diabetic, traumatic, burn, and surgical wounds [1,2,3,4]. Extending this concept, it can be understood that the closed-incision negative-pressure wound therapy (iNPWT) system has been proven to enhance wound healing by holding the incision wound edge together, removing fluid and preventing contamination, increasing local blood perfusion, reducing tissue edema, decreasing lateral stress on the incision by approximately 50%, reducing and normalizing tissue stress around the incision shearing force at the suture line, and increasing lymphatic drainage [5,6,7]. It has been applied in aesthetic surgeries, such as abdominoplasty, to enhance wound healing, reduce postoperative fluid accumulation, and remove drains earlier [8]. Its application with various incisions also presented promising results [9]. iNPWT has also been applied to the surgical wounds of abdominoperineal resections to prevent wound infections [10]. Zwanenburg et al. further support the effect of iNPWT, not only on wound healing, but also on surgical site infection [11].

Despite clinical experience, the information regarding the mechanism of iNPWT with incisions under tension is limited.

**Figure 1:**
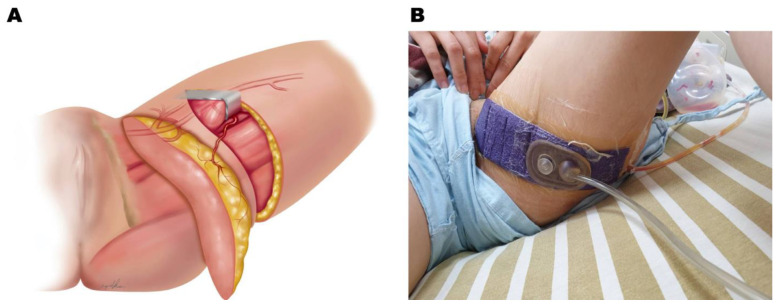
Flap design and clinical application of iNPWT. (**A**) A sketch illustrates how the flap is designed and harvested. (**B**) Postoperative application of closed-incision negative pressure on the donor site of a PAP free flap harvested in the transverse manner.

**Figure 2:**
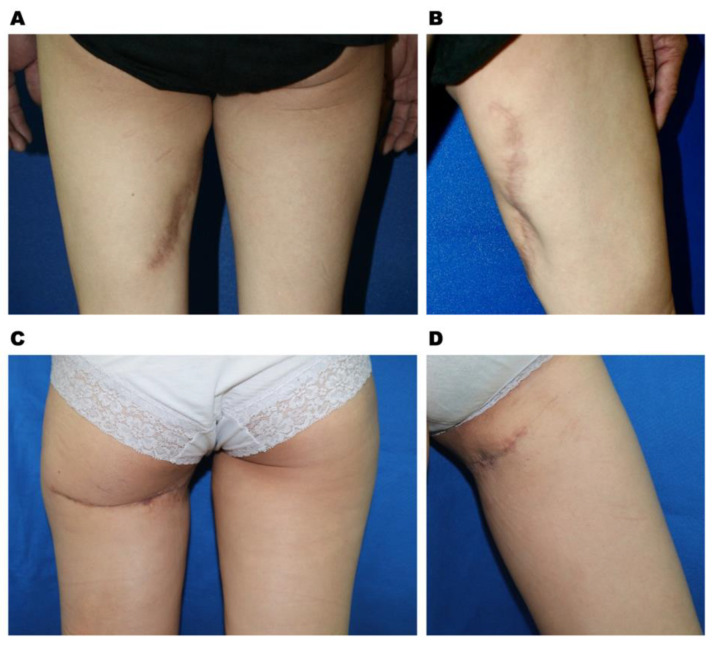
Donor site appearance after a PAP flap is harvested with a vertical flap design in the posterior (**A**) and anterior medial (**B**) view at follow-up session at 22 months and with transverse flap design in posterior (**C**) and anterior medial (**D**) view at follow-up session at 21 months.

Among alternative flaps other than deep inferior epigastric artery perforator (DIEP) free flaps used for breast reconstruction, the profunda artery perforator (PAP) free flap is emerging as a second option. Sharing a similar anatomical site with transverse upper gracilis myocutaneous (TUG) flaps, the PAP flap is located slightly more caudally than the transverse flap design, eliminating the complication of distortion of the labia major (Figure 1A). The PAP free flap was traditionally designed with a transverse scar below the gluteal line. Various modifications of the flap design have been proposed to enhance wound healing and increase flap volumes, such as longitudinal designs, fleurs-de-lis, or S scars [12,13,14]. Figure 2 demonstrates two cases of donor sites after PAP harvesting, one via a transverse design and the other via a vertical design. The transverse flap design remains a good choice for the senior author due to the aesthetic consideration of hiding the scar. Due to the counter direction of muscle contractions during walking, however, the transverse flap design increases the tension on the incision and the risk of poor wound healing, wound tearing, infection, ischemic necrosis, or dehiscence [15,16]. Different suture and modified techniques have been developed to reduce tension during wound closure [17,18,19]. In addition, the emerging application of negative pressure can offer adjuvant management.

Negative pressure has been widely applied to open wounds or closed incisions. For closed-incision wounds, clinically promising results have been reported. The mechanism of its role in closed incisions has been studied using a porcine model with a regular incision and closure. Its role in closed incisions under tension, however, has not been well explored in the literature. In this study, the mechanism of iNPWT on tension wounds was first explored in a porcine model with the incision closure under tension and further applied in a transversely designed donor site of a PAP flap that was under tension and stretching following ambulation. Clinically, in order to minimize the complications from a closed incision under tension, patients receiving PAP free flaps for breast reconstruction were offered the iNPWT for their donor sites (Figure 1) and the results were evaluated.

## 2. Materials and Methods

### 2.1. Animal Research on Negative Pressure Treatment of Tension Wounds in a Porcine Model

**Figure 3:**
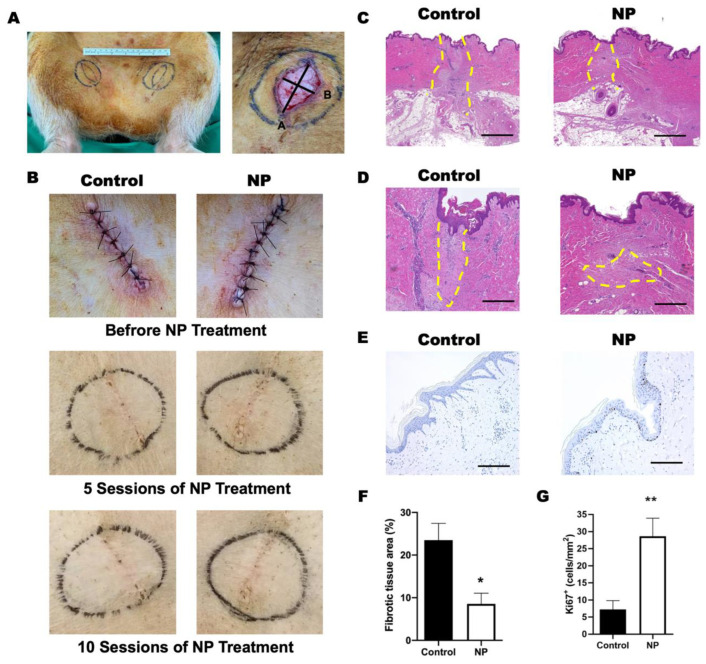
Negative-pressure (NP) treatment in a porcine model. (**A**) Left: the design of the fusiform excision in the bilateral groin area to create wound tension. Right: the gross appearance of the wound after excision, which was fusiform and sized 2.5 cm in width and 3.5 cm in length. (**B**) The gross view of wound area before and after 5 and 10 sessions of negative-pressure treatment. (**C**) The granulation tissue (within the yellow dashed line) was observed in both the control and NP groups. (**D**) The magnification view of the granulation tissue in (**C**). Scale bar: 500 µm. (**E**) The proliferated keratinocytes are marked with Ki67 (brown). Scale bar: 200 µm. (**F**) The quantification of the fibrotic tissue area. (n = 4) (**G**) Quantification of Ki67^+^ cells per mm^2^ (n = 4). * *p* < 0.05, ** *p* < 0.01. Error bar: standard error.

All of the animal experiments were performed under the guidelines of the animal center of Chang Gung Memorial Hospital (IACUC# 2018082801). Four Lanyu miniature pigs (15 to 20 kg in body weight) were used. After fasting for 8 to 12 h, the miniature pigs were injected with atropine (atropine sulfate injection, Sinttong Taiwan) intramuscularly, followed by a tiletamine and zolezepam 1:1 mixture (Zoletil50, Virbac, France) 0.55 to 0.80 mg/kg intramuscularly to achieve the initial stage of anesthesia. Gas anesthesia with isoflurane 2.5% to 4.5% was used for the induction, followed by 1% to 3% in maintenance after the completion of intubation. Following the anesthesia, the pigs were placed in the supine position, and the skin in the groin region was prepared in an aseptic manner. A fusiform (2.5 cm in width and 3.5 cm in length) incision was created on both sides of the groin region (Figure 3A) and closed with simple suturing using 4-0 Nylon sutures. A custom-manufactured negative-pressure device was placed on one side of the groin wound to cover the whole wound as iNPWT treatment, leaving the other side as a control. Due to the difficulty of maintaining continuous negative pressure on the animal for seven days, the application of negative pressure was modified as follows. A pressure of −70 mmHg was applied to the incision for three hours per treatment and twice per week for five weeks. The suction cups were directly applied to cover the closed wound. No dressing or sponge were used under the suction device. After five weeks, the scars over the groin area, including 1 cm on each side, were harvested in a full-thickness manner, including the subcutaneous tissue, for further analysis [20].

### 2.2. Histological Evaluation

Hematoxylin and eosin (H&E) staining was performed to visualize the morphological changes in the skin tissue. The images were processed by a Leica DMRXA microscope (Leica Microsystems Ltd., Wetzlar, Germany) using Image-Pro Premier software, version 9.0 (Media Cybernetics, Inc., Rockville, MD, USA). The fibrotic tissue area was measured as follows:

Fibrotic tissue area (%) = Fibrotic tissue area/Total tissue area × 100%

### 2.3. Immunohistological Staining and Evaluation

The samples were fixed with 4% paraformaldehyde (100496, Sigma, St. Louis, MI, USA) and embedded in paraffin. Tissue blocks were sectioned at a 4 µm thickness for the histological analysis. Slides were rehydrated in PBS and blocked with blocking solution (1.5% BSA, A9418, Sigma, St. Louis, MI, USA; PBS, 70011, GIBCO, Billings, MT, USA) prior to overnight incubation. The whole-mount samples were incubated with primary antibodies against Ki67 (ab16667, 1:100 solution: Abcam, MA, USA), collagen I (ab90395, 1:200 solution: Abcam, MA, USA), CD31 (ab28364, 1:100 solution; Abcam, MA, USA), *α*SMA (ab7817, 1:200 solution: Abcam, MA, USA), keratin 16 (ab76416, 1:100 solution: Abcam, MA, USA), CD3 (ab16669, 1:200 solution: Abcam, MA, USA), CD4 (ab133616, 1:200 solution: Abcam, MA, USA), CD8 (ab4055, 1:200 solution: Abcam, MA, USA), CD45 (ab8216, 1:200 solution: Abcam, MA, USA), and CD68 (ab9551:200 solution: Abcam, MA, USA) for 2 h in the dark prior to incubation with a secondary antibody (ab150077, ab150080, ab150113, ab150116, 1:500 dilution; Abcam, MA, USA) for 24 h. After three washes, the slides were coverslipped and viewed with a TCS-SP8X (Leica, Wetzlar, Germany) confocal microscope in the Microscopy Core Laboratory, Chang Gung Memorial Hospital. The stained sections were digitally imaged with an Axio Scope A1 microscope (Carl, Zeiss, Jena, Germany) and later subjected for analysis. In each view area (5× objective, 1.4 mm × 1 mm area), the marker-labeled area or cells were measured and counted, respectively. For each tissue section, five view areas were randomly selected and quantified.

### 2.4. RNA Isolation and Quantitative Real-Time PCR (RT–PCR)

The samples were mixed with chloroform to extract RNA. Subsequently, the RNA was reverse transcribed into cDNA using a high-capacity cDNA reverse transcription kit (4368813, Applied Biosystems, Foster City, CA, USA) following the instructions provided by the suppliers. Primers were designed and customized by GeneDireX (USA). The sequences are presented in Table 1. Then, quantitative real-time PCR was performed using a StepOnePlus RT–PCR machine (Applied Biosystems, Foster City, CA, USA). The quantity of cDNA in each sample was normalized to the crossing point of the housekeeping gene β-actin.

### 2.5. Clinical Application of Negative Pressure at the Donor Site of the PAP Flap

After obtaining permission from the institutional review board (#202101649B0), a retrospective chart review was conducted to identify patients receiving unilateral microsurgical breast reconstruction from the senior author (JJH) from November 2016 to August 2020. By excluding patients with delayed reconstruction who required a larger skin paddle for reconstruction, a total of 25 patients were identified who fulfilled our inclusion criteria, including two delayed immediate reconstructions with previously implanted tissue expanders. The patients were separated into two groups according to the application of iNPWT at the donor site (Figure 1A,B). Patient demographic data, including their age at surgery, body mass index (BMI), medical comorbidities, including hypertension and diabetes, and smoking histories, were obtained from the retrospective chart review. The parameters of the flap were carefully reviewed, including the flap harvested width and length, the flap harvested and used size, the size of the mastectomy, and the ischemia time of the flap.

The complications of the donor site were categorized into acute and chronic, depending on the time of presentation. Donor site complications, including wound breakdown, hematoma and seroma, and the total amount of drainage were recorded. The cutting time point for acute and chronic complications was one month. The total amount of drainage from the closed suction drain and the patients’ off-bed time (day) was recorded. For patients with a follow-up time of longer than one year, the scar was evaluated using the Vancouver scar scale [21].

### 2.6. Application of iNPWT

All donor sites were primarily closed with a suction drain. For patients in the iNPWT group, iNPWT was applied immediately following wound closure (PREVENA Incision Management System, KCI, an Acelity company, San Antonio, TX, USA) with a pressure of 125 mmHg in continuous mode for 7 days.

### 2.7. Statistical Analysis

The data were analyzed using SPSS graphing and statistical analysis software, version 21 (IBM, Chicago, IL, USA). The rank sum and independent *t* tests were applied to calculate the continuous variables in the demographic values. The nonparametric *t* test and the Mann–Whitney test were also performed to compare collagen deposition, the level of inflammation, the level of angiogenesis, and the number of LYVE-1 and podoplanin-labeled cells between the control and negative-pressure (NP) groups. A probability of less than 0.05 was considered significant.

## 3. Results

### 3.1. Negative Pressure Enhanced Wound Healing under Tension in the Animal Study

All of the wounds healed primarily without infection or dehiscence in both groups. The skin color and texture were grossly similar after 5 and 10 NP treatments between groups (Figure 3B). Complete epithelialization could be observed by H&E staining in all except one pig from the control group. Following the semiquantitative evaluation of the histological parameters for the assessment of wound healing [22], the features are presented in Table 2. The skin tissue obtained from the control group contained a multilayer of epithelium on the wound edge, suggesting incomplete epithelialization.

Immature granulation tissue containing macrophages and sparse extracellular matrix was observed in both groups. The level of immature granulation tissue in the control group was considered moderate (present in three of four samples), whereas slight immature granulation was present in the NP group (present in three of four samples) (Figure 3C,D,F). The result indicates that the healing process is not completed in either group within 5 weeks. In addition, the epidermis in the NP group was significantly thicker with significantly increased numbers of Ki67^+^ cells (control vs. NP: 7.3 ± 2.6 vs. 29 ± 5.3 cells/mm^2^, *p* < 0.01), suggesting stronger epithelium formation and more cellular differentiation (Figure 3E,G). Along with better epithelialization, significantly less fibrosis was observed in the NP group (control vs. NP: 24 ± 3.9% vs. 8.6 ± 2.5%, *p* = 0.012) (Figure 3F).

### 3.2. Negative Pressure Enhanced Collagen Deposition in the Animal Study

**Figure 4:**
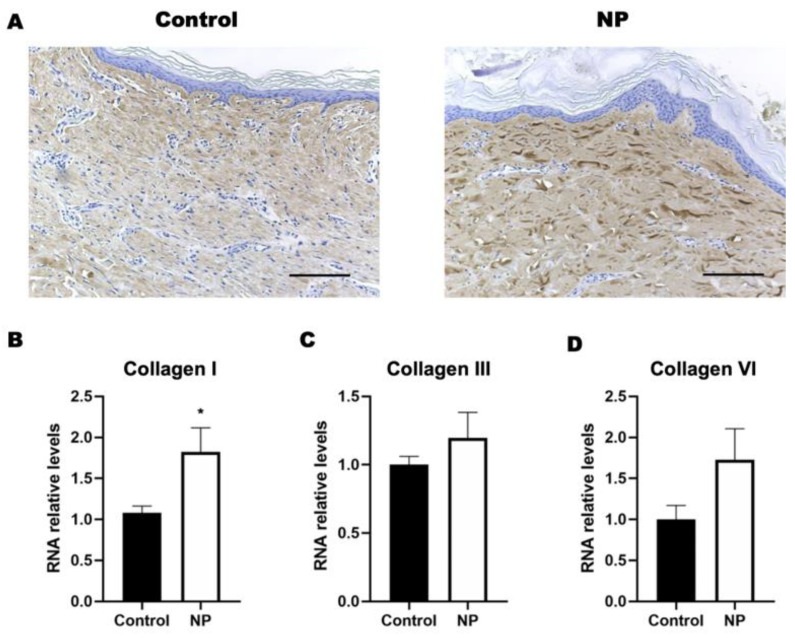
The collagen expression in porcine skin tissue following negative-pressure treatment. (**A**) Histological staining of collagen type I in the control and NP groups. (**B**–**D**) The gene expression of collagens I, III, and VI in the control and NP groups (n = 4). Scale bar: 200 µm. * *p* < 0.05. Error bar: standard error.

The expression of collagen type I significantly increased (Figure 4A) in response to the negative-pressure treatment, which was further confirmed by the RNA expression of collagen I (control vs. NP: 1.1 ± 0.08 vs. 1.8 ± 0.2, *p* = 0.02). Unlike the presentation of collagen type I, the presentation of collagen types III or IV did not present a difference between the NP and control groups (Figure 4B–D).

**Table 2:** The assessment of histological parameters for wound healing.

	Control	NP Treatment
Epidermal regeneration	++ (3/4)	+++ (4/4)
Granulation tissue	++	+
Inflammatory cell infiltration	++	+
Angiogenesis	+	+++
Fibrotic tissue	++	+
Collagen deposition	++	+

Note: + slight, ++ moderate, +++ extensive. NP, negative pressure.

### 3.3. Negative Pressure Promoted Angiogenesis in the Animal Study

**Figure 5:**
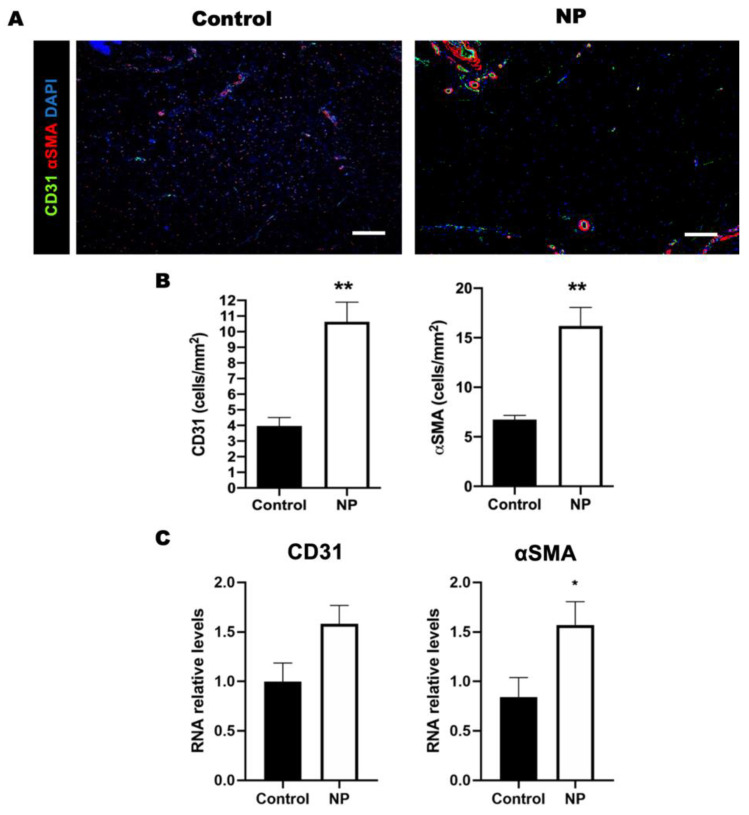
The level of angiogenesis in the wound area is elevated after negative-pressure treatment. (**A**) Immunohistological staining of CD31 (green) and αSMA (red) in the control and NP groups. (**B**) The density of blood vessels labeled with CD31 and αSMA is higher in the NP group. (**C**) The RNA expression of CD31 and αSMA in the control and NP groups (n = 4). Scale bar: 200 µm. * *p* < 0.05, ** *p* < 0.01. Error bar: standard error.

The significantly increased presentation of CD31 (control vs. NP: 4 ± 0.53 vs. 11 ± 1.3 CD31-labeled cells/mm^2^, *p* < 0.001) and αSMA (control vs. NP: 6.7 ± 0.41 vs. 16 ± 1.9, *p* < 0.001) in the NP-treated wound tissue suggested the enhancement of angiogenesis by treatment using NP (Figure 5A,B). Similarly, the RNA expression of CD 31 (control vs. NP: 1 ± 0.19 vs. 1.6 ± 0.19, *p* = 0.05) and αSMA (control vs. NP: 0.84 ± 0.2 vs. 1.6 ± 0.23, *p* = 0.03) was significantly higher in the tissue sample obtained from the NP group (Figure 5C). The correlation of tissue presence with RNA expression suggested that angiogenesis was promoted by negative-pressure treatment.

### 3.4. Negative Pressure Treatment Decreased the Inflammatory Response in the Animal Study

**Figure 6:**
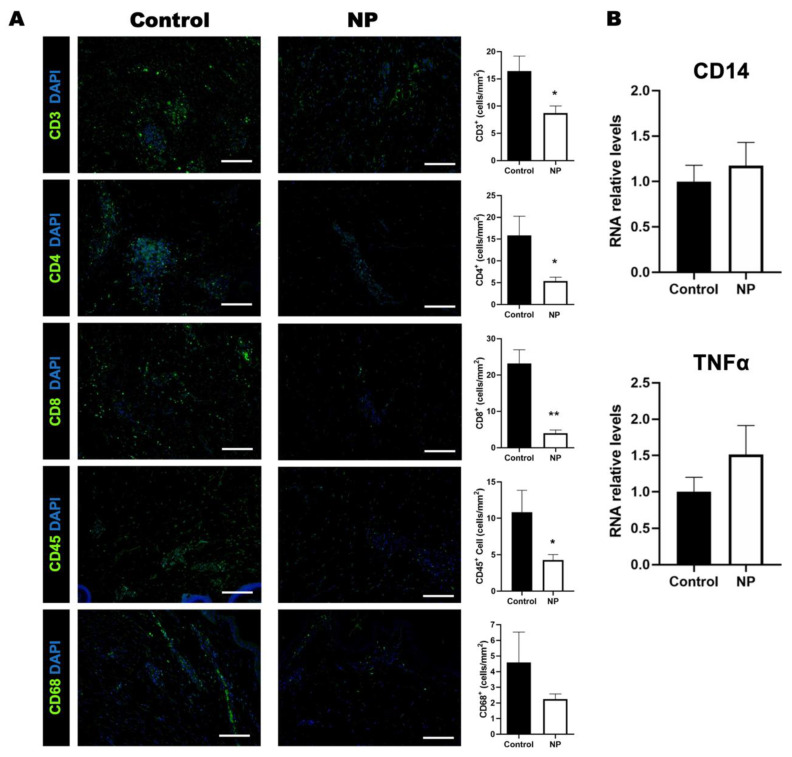
The inflammatory cells reduced following negative-pressure treatment. (**A**) Immunohistological staining of CD3, CD4, CD8, CD45, and CD68 (green) in the control and NP groups. The quantification of CD3, CD4, CD8, CD45, and CD68 expressions in the control and NP groups. (**B**) The RNA expression of CD14 and TNFα in the control and NP groups (n = 4). Scale bar: 200 µm. * *p* < 0.05, ** *p* < 0.01. Error bar: standard error.

Immunofluorescent staining of the specimens revealed the reduced infiltration of CD3^+^ (*p* = 0.02), CD4^+^ (*p* = 0.03), CD8^+^ (*p* < 0.01), and CD45^+^ (*p* = 0.04) cells in the NP-treatment group compared to the control group (Figure 6A,B). The infiltration of CD 68^+^ cells was similar between the two groups (*p* = 0.25) (Figure 6). The RNA expression of another macrophage marker (CD14) (control vs. NP: 1.0 ± 0.18 vs. 1.2 ± 0.26, *p* = 0.58) also showed no significant increase in RNA expression. The expression of the proinflammatory cytokine tumor necrosis factor alpha (TNF*α*) in the NP group did not differ significantly from that of the control group (control vs. NP: 1.0 ± 0.12 vs. 1.5 ± 0.4, *p* = 0.27) (Figure 6B).

### 3.5. Negative Pressure Treatment Enhanced Lymphangiogenesis in the Animal Study

**Figure 7:**
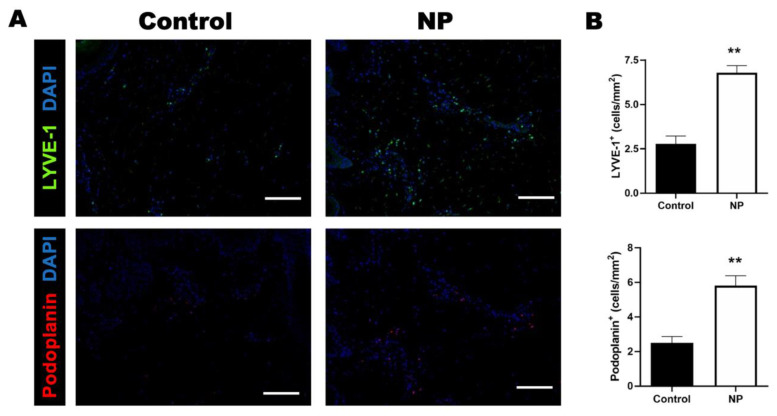
The numbers of LYVE-1 and podoplanin cells increased in the NP group. (**A**) Immunohistological staining of LYVE-1 (green) and podoplanin (red) in the control and NP groups. (**B**) The quantification of LYVE-1- and podoplanin-labeled cells in the control and NP groups (n = 4). Scale bar: 200 µm. ** *p* < 0.01. Error bar: standard error.

Negative pressure promotes healing in open wounds by enhancing the drainage of excess body fluid [23]. Whether negative pressure enhances the drainage function by promoting lymphangiogenesis is worth investigating. The number of lymphatic vessel endothelial hyaluronan receptor 1 (LYVE-1)- and podoplanin-stained cells were significantly increased following negative-pressure treatment, suggesting that negative pressure promotes lymphangiogenesis in a closed incision under tension (the number of LYVE-1-stained cells: control vs. NP: 2.8 ± 0.44 vs. 6.8 ± 0.41, *p* < 0.01; the number of podoplanin-stained cells: control vs. NP: 2.5 ± 0.37 vs. 5.8 ± 0.57, *p* < 0.01) (Figure 7A,B).

### 3.6. Negative Pressure Treatment in the Clinical Study

**Figure 8:**
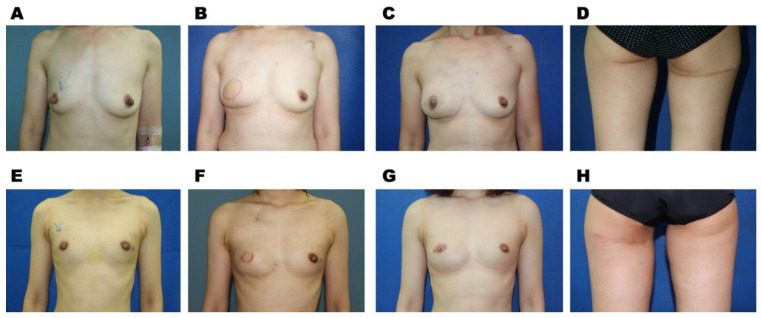
Clinical examples. (**A**) A preoperative anterior posterior view of a 35-year-old female patient with right-breast cancer. Following a skin-sparing mastectomy, reconstruction was performed using a PAP free flap obtained from her right thigh. (**B**–**D**) Follow-up photos of the 35-year-old female patient. (**B**) Postoperative appearance at her follow-up session of seven months. (**C**) Post-nipple areolar reconstruction at the follow-up session of 21 months from the mastectomy. (**D**) The donor-site appearance 21 months following surgery. (**E**) A preoperative anterior posterior view of a 30-year-old female patient with right-breast cancer. Her postoperative photos are shown in (**F**–**H**). (**F**) Postoperative anterior posterior view of the patient following a skin-sparing mastectomy and PAP free flap reconstruction at the follow-up session of four months. (**G**) Postoperative photo taken following nipple–areolar reconstruction at the 27-month follow-up session. (**H**) The donor-site scar over her left thigh at the follow-up session of nine months.

A total of 25 patients were included in the study, with 12 in the NP group and 13 in the control group. None of the patients included had other medical comorbidities or smoking histories. Both groups shared similar demographic data and the same breast cancer characteristics (Table 3). Figure 8 illustrates a case example of both breast reconstruction and donor site appearance at the one-year postoperative follow-up session.

The flap harvested weight and flap used weight were significantly lower in the NP group than in the control group (flap harvested weight: 224.9 ± 59.5 and 293.4 ± 87.9 g in the NP and control groups, respectively, *p* = 0.034, and flap used weight: 209.3 ± 55.2 and 271.9 ± 78.5 g in the NP and control groups, respectively, *p* = 0.032). Considering the ratio of the flap used weight and the mastectomy size, the ratio was 93.7 ± 27.7% in the NP group and 107.4 ± 42.1% in the control group. There was a relatively inadequate replacement of the mastectomy volume in the NP group, suggesting that the patients in the NP group tended to have an inadequate flap size, and as a result, the replacement of the resected breast was relatively inadequate despite harvesting the flap to its maximal volume. Although the difference did not achieve a significance, the remaining parameters of the flaps, including the flap width and length, the length of the pedicle, the included perforator numbers, and the mastectomy size, were compatible between the two groups (Table 4).

The postoperative course and complications are presented in Table 5. The major difference was presented in the off-bed time, which was on the 4.6 ± 1.1st and 5.5 ± 0.8th postoperative days in the NP and control groups, respectively (*p* = 0.028). No acute complications presented at the donor site of the NP group, while there were three in the control group, including two patients with wound breakdowns that required surgical debridement and one with a wound breakdown that healed secondarily following wound care. The total drainage amount of the drain at the donor site was 156.9 ± 57.3 and 166.8 ± 62.9 mL for the NP and control groups, respectively (*p* = 0.701), and the day of drainage tube removal was 7.7 ± 1.5th and 8.4 ± 1.4th postoperative days in the NP and control groups, respectively (*p* = 0.269). A smaller drainage volume and earlier drainage removal were noted in the NP group, but the difference was not statistically significant.

Four patients in the control group presented with late complications, including three wound breakdowns (one required surgical debridement and closure, and two healed spontaneously following wound care) and one donor seroma. One patient in the NP group experienced donor-site wound breakdown that healed secondarily. Thus, although the late complications tended to be greater in the control group (30.8% and 8.3% in the control and NP groups, respectively, *p* = 0.3217), the difference was not significant. Two (16.7%) and three (23.1%) patients in the NP and control groups underwent scar revisions. None of our patients presented with transient or permanent lower-extremity lymphoedema or the distortion of the labia majora. For the patients with follow-up sessions of more than one year (13 in the control group and 8 in the NP group), the donor-site scar was evaluated using the Vancouver scar scale, which did not present a difference between the two groups (Table 5).

## 4. Discussion

The application of the PAP flap in breast reconstruction dates back to 2012, when Allen and colleagues first transferred PAP flaps in patients without adequate abdominal tissue for breast reconstruction [24,25]. For aesthetic reasons, the flap was first designed in a transverse manner to hide the scar below the gluteal line [24]. Despite the fact that that some revisional designs were later proposed to increase tissue volume or reduce complications from a transverse incision, the transverse incision was preferred by the senior author (JJH) for aesthetic reasons [12,13,14,26]. Many of the complications of the donor site of a PAP flap were referred to the transverse flap design. Cho and colleagues revised their approach towards transverse flap design with a more conservative inclusion of flap height, beveling to include higher flap volume, using longer acting absorbable sutures, having the patients wear compression garments after surgery, and applying posterior thigh advancement flaps for wound closure if needed [27]. Tension as one of the predisposing factors for poor wound healing is encountered largely in the transverse design of the PAP flap for breast reconstruction. Following primary wound healing, the healed wound is an early scar under even greater tension when the patients are allowed to fully ambulate. The iNPWT maintains a stronger tissue approximation on the closed incision during the early wound healing phase [28]. Negative pressure not only enhances the wound healing process, but also the tissue strength, which reached a level similar to that of normal skin [28]. Maintaining the mechanical properties of the healed tissue is equally important to maintain the tissue strength for resistance to late wound breakdown and subsequent infection. This process is especially important in clinical practice.

The issue of tension release by negative pressure has been addressed in basic research. Wilkes et al. presented a 55% reduction in lateral tension as key to maintaining the integrity of closed wounds by applying iNPWT to closed incisions [6]. An increase in skin tension results in the expansion or elongation of the extracellular matrix (ECM) and cells [29]. Unlike most of the available animal studies, in which the closed incision is not under tension, we created a closed-incision wound in the groin area of pigs by removing a piece of tissue to induce tension on wound closure. Our purpose was to shed further light on the role of iNPWT to include the effect of tension and mimic clinical demands. Our approach resulted in a closed incision that was under tension; in particular, the animals were allowed full ambulation. The results of the animal study are in accordance with the issue of expansion or elongation of the ECM by showing a drastically increased number of cells expressing Ki67 under negative pressure. This suggests that the cell proliferation of keratinocytes, in particular, in the epidermal layer might be a possible responsive pathway to release wound tension.

The scar evaluation score was found to be higher in closed wounds treated with negative pressure in the animal study, and increased vascular endothelial growth factor (VEGF) expression was also observed [30]. A previous publication also proved that negative pressure promoted the expression of vascular endothelial growth factor receptors (VEGFRs) in an NPWT wound healing rabbit model of open wounds [31]. Furthermore, negative-pressure treatment for wound healing resulted in vessel destabilization and maturation by decreasing the expression ratio of angiogenin 1 (Ang1)/Ang2 [32]. In our study, enhanced angiogenesis was noted after treatment with iNPWT with evidence showing a significantly higher expression of both endothelial cell and vascular wall smooth muscle cell markers (CD31 and αSMA). This confirms that the angiogenesis was enhanced not only in open wounds, but also in closed incisions with the treatment of negative pressure.

In addition to tension release, the increase in inflammation and scar formation by mechanical stress [33] could be relieved under the iNPWT approach [34]. The reduction in inflammation was presented in our results with significantly less CD3^+^, CD4^+^, CD8^+^, and CD45^+^ cell infiltrations. Inflammation delays the process of wound healing, and chronic inflammation can even result in nonhealing wounds [35]. The results obtained from a microarray analysis performed on stretched and non-stretched wound tissues suggested that the mechanical force elevated T-cell-dependent Th2 cytokine signaling in stretched wound tissue [36]. In T-cell-deficient mice, scar formation was reduced nine-fold [36]. These studies indicated that the stretched forces elevated the inflammatory response during wound healing and led to scar formation. Our results indicate that the infiltration of inflammatory cells is significantly reduced under negative-pressure treatment on closed-incision wounds under tension. Considering the results together, iNPWT treatment promoted closed-incision wound healing in multiple ways. It enhanced angiogenesis and lymphangiogenesis. It reduced the inflammatory reaction; the mechanical force that was reduced under negative pressure further contributed to the reduction in inflammation. In addition to the enhancement of wound healing, scar formation was also better controlled.

In our clinical study, our results suggest that the application of iNPWT in the transversely designed donor incision of a PAP free flap is likely to improve wound healing and reduce complications. A significant difference was only present in the reduction in bed-rest time (determined by the flap observation time of three days plus the pain that limited the patient’s movements). Fewer complications were noted at the donor site when iNPWT was applied, but the difference was not enough to achieve significance. Of note, the harvested flap weight was significantly lower in the NP group; similarly, the flap weight used for reconstruction was significantly lower in the NP group. However, an equal size of the flap harvested for breast reconstruction is sometimes not possible due to individual differences. Additionally, harvesting a PAP flap with the same flap width and length can still result in different wound tension after closure since the body stature is different. Although we always harvested a flap as wide and as large as possible, we could only obtain a smaller flap in the NP group. As a result, the ratio of the flap used weight and the mastectomy weight was lower in the NP group, and a relatively inadequate replacement of the mastectomy weight was noted in the NP group (non-significant). Although the difference of the replacement was not significant, we can assume that the flap harvested in the NP group already achieved its maximum value, and that the tension experienced during donor-site closure could be even greater.

**Figure 9:**
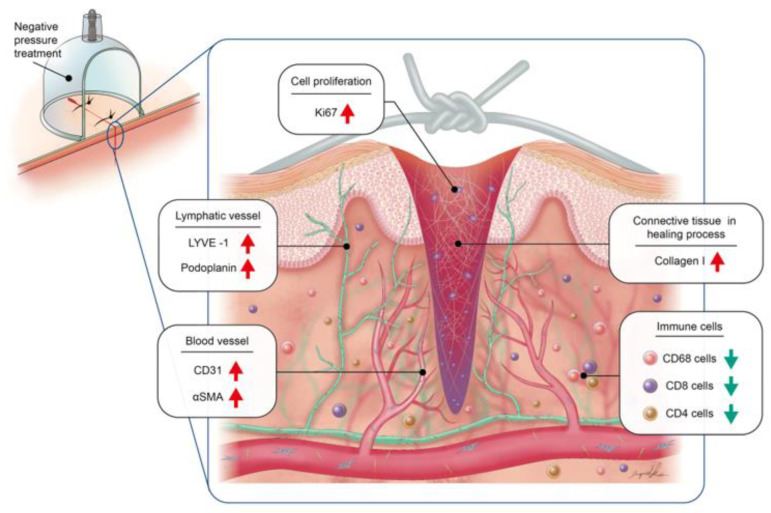
Schematic illustration of the effect of negative pressure on wound healing.

The histological features obtained from our animal study confirmed better tissue-regeneration results in the NP group. Our experience presented convincing results in the application of negative pressure on closed-incision wounds under tension. Despite the promising results, several limitations exist. First, the patient number remained small, and the patient selection between iNPWT was not random (we started the application of iNPWT after the device was available in our center). Considering the medical cost of an iNPWT system and the benefits from the use of it (off-bed time was one day shorter and non-significantly fewer acute and late complications were present), further prospective studies with similar patient selection criteria can be conducted. Moreover, many of the reported animal studies used swine models with surgical wounds created in the dorsum of the animal. Within the literature [28], our approach of creating a tension wound in the groin area contributed further information about the healing of a tension wound with the assistance of negative pressure. With the limitations of performing this on animals, constant (24 h) negative-pressure treatment in a porcine model is not applicable. However, since the animals moved without restrictions, we could observe the effect of negative pressure on a tension wound, which was dynamically unfavorable for early ambulation. In our study, applying negative pressure on high-tension wounds resulted in better wound healing outcomes through the promotion of keratinocyte proliferation and angiogenesis, a reduction in the inflammatory response, and an increase in lymphatic vessel density (Figure 9). This evidence supports the clinical application of negative pressure on high-tension wounds and provides an alternative approach to wound healing procedures.

## 5. Conclusions

In conclusion, the application of iNPWT to closed-incision wounds under tension effectively enhanced the wound healing process with earlier recovery results. The evidence and mechanism of iNPWT in closed-incision wounds under tension were confirmed by an animal study with the enhancement of angiogenesis and lymphangiogenesis, and greater collagen deposition with reduced inflammatory reactions.

## Figures and Tables

**Table 1:** Primer sequence.

Gene Name	Forward Primer	Reverse Primer
β-Actin	CGCGAGAAGATGACCCAGAT	GGAGGGCATACCCCTCGTAG
Collagen I	GACCTGCGTGTACCCCACTC	TGGGAAGCCTCAGTGGACAT
Collagen III	AGAGGAGTTGCCGGAGAACC	TTCCAGGAGCACCGTCATTT
Collagen VI	GCACTCCGGGAGGTACACAG	GGTCTCCTCGGACACCCTCT
CD31	TTCTCCTGAGCGAGGAGGTG	CAAATGGCCTGGGTGTCATT
*α*SMA	GAACACGGCATCATCACCAA	GGACAGCACCGCCTGAATAG
CD14	AACTGACGCTCGAGGACCTG	TGCTTGCGCCACTTTCAGTA
TNF*α*	TGTGCCTCAGCCTCTTCTCC	TGTCCCTCGGCTTTGACATT

**Table 3:** Clinical characteristics and demographics of two groups of patients.

	Control Group	NP Group	*p* Value
	n = 13	n = 12	
Age (years)	39.2 ± 8.9	38.2 ± 5.5	0.746
BMI (kg/m^2^)	21.8 ± 3.2	20.4 ± 1.1	0.145
Smoking	0	0	
Hypertension	0	0	
Diabetes mellitus	0	0	
TNM staging			
Stage 0 (DCIS)	3 (23.1%)	7 (58.3%)	0.092
Stage I	3 (23.1%)	4 (33.3%)	
Stage II	4 (30.8%)	1(8.3%)	
Stage III	3 (23.1%)	0 (0%)	
ER status			
Positive	8 (61.5%)	6 (50%)	0.561
Negative	5 (38.5%)	6 (50%)	
PR status			
Positive	8 (61.5%)	6 (50%)	0.561
Negative	5 (38.5%)	6 (50%)	
HER-2 status			
Positive	9 (69.2%)	9 (75%)	1.000
Negative	4 (30.8%)	3 (25%)	

Data are presented as n (%) or mean ± standard deviation. NP, negative pressure; BMI, body mass index; ER, estrogen receptor; PR, progesterone receptor; HER-2, human epidermal growth factor receptor-2.

**Table 4:** Flap details.

	Control Group	NP Group	*p* Value
Variables	n = 13	n = 12	
Flap width (cm)	7.7 ± 1.3	7.8 ± 0.9	0.895
Flap length (cm)	21.2 ± 3.6	20.5 ± 2.9	0.585
Pedicle length (cm)	5.7 ± 1.5	5.8 ± 1.5	0.817
Perforator numbers	1.6 ± 0.7	1.4 ± 0.7	0.459
Harvest weight (g)	293.4 ± 87.9	224.9 ± 59.5	0.034 *
Flap used weight (g)	271.9 ± 78.5	209.3 ± 55.2	0.032 *
Flap used (%)	93.3 ± 6.8	93.1 ± 4.0	0.958
Mastectomy weight (g)	264.8 ± 99.8	236.6 ± 99.1	0.505
Flap used weight/mastectomy weight (%)	107.4 ± 42.1	93.7 ± 27.7	0.371

Data are presented as mean ± standard deviation. NP, negative pressure. * *p* < 0.05.

**Table 5:** Comparative results between control and negative-pressure wound therapy groups.

	Control Group	NP Group	*p* Value
Variables	n = 13	n = 12	
Off-bed time (days)	5.5 ± 0.8	4.6 ± 1.1	0.028 *
Drainage amount (mL)	166.8 ± 62.9	156.9 ± 57.3	0.701
Vacuum ball removed timing (post-op days)	8.4 ± 1.4	7.7 ± 1.5	0.269
Vancouver scar scale	5.3 ± 2.9	5.8 ± 2.5	0.693
Re-exploration	2 (15.4%)	1 (8.3%)	1.000
Donor site acute complications (<30 days)	3 (23.1%)	0	0.22
Wound breakdown required surgery	2	0	
Wound dehiscence with wound care	1	0	
Donor site long-term complications (>30 days)	3 (23.1%)	1 (8.3%)	0.593
Wound breakdown required surgery	1	1	
Wound dehiscence with wound care	2	0	
Donor-site revision	3 (23.1%)	2 (16.7%)	1.000
Lower limb lymphedema	0	0	
Distortion of major labia	0	0	

Data are presented as n (%) or mean ± standard deviation. NP, negative pressure. * *p* < 0.05.

## Data Availability

The data used and analyzed during the current study are available from the corresponding author upon request.
